# Icariin inhibits the inflammation through down-regulating NF-κB/HIF-2α signal pathways in chondrocytes

**DOI:** 10.1042/BSR20203107

**Published:** 2020-11-23

**Authors:** Pengzhen Wang, Qingqi Meng, Wen Wang, Shaoheng Zhang, Xifeng Xiong, Shengnan Qin, Jinli Zhang, Aiguo Li, Zhihe Liu

**Affiliations:** 1 Guangzhou Institute of Traumatic Surgery, Guangzhou Red Cross Hospital, Jinan University, Guangzhou, Guangdong 510220, China; 2 Department of Orthopaedics, Guangzhou Red Cross Hospital, Jinan University, Guangzhou, Guangdong 510220, China; 3 Department of Cardiology, Guangzhou Red Cross Hospital, Jinan University, Guangzhou, Guangdong 510220, China

**Keywords:** chondrocyte, Icariin (ICA), inflammation, NF-κB/HIF-2α

## Abstract

Articular cartilage injury or defect is a common disease and is mainly characterized by cartilage degradation because of chondrocyte inflammation. By now, there are no effective drugs and methods to protect articular cartilage from degradation. Icariin (ICA) is a typical flavonoid compound extracted from *Epimedii Folium* with anti-inflammatory and bone-protective effects. Our previous studies demonstrate that ICA up-regulates HIF-1α expression and glycolysis in chondrocytes and maintains chondrocyte phenotype. As another member of HIFs family, HIF-2α always plays a key role in inflammation. The effect of ICA on HIF-2α is unclear by now. In the present study, we confirmed the findings in our previous study that ICA promoted not only chondrocyte vitality and extracellular matrix (ECM) synthesis, but also the anti-inflammatory effect of ICA. In bone defect mice, ICA inhibited the expressions of NF-κB and HIF-2α. In TNF-α-treated ADTC5 chondrocytes, ICA neutralized the activation of IKK (IKK phosphorylation), the phosphorylation of IkB and NF-κB and the expression of HIF-2α. Furthermore, ICA inhibited the nucleus transfer of NF-κB and the expressions of MMP9 and ADAMTS5, two key targets of NF-κB/HIF-2α signal pathway. Taken together, the present study demonstrated that ICA may increase the vitality of chondrocytes by suppressing the inflammatory injury through the inhibition on NF-κB/HIF-2α signaling pathway. ICA is one effective candidate drug for the treatment of articular cartilage injury.

## Introduction

Articular cartilage is a highly organized avascular connective tissue with limited regenerative ability following trauma or degenerative pathology [1]. Articular cartilage injury is a common disease that occurs in major joints such as knee and hip joints [2]. The pathological features of cartilage damage are chondrocyte apoptosis, aging and the reduction of extracellular matrix (ECM) synthesis. SOX9, metalloproteinase (MMPs) and aggrecanase are key regulators of ECM metabolism [3]. In addition to other considerations such as aging, common cartilage injuries are caused by mechanical traumas [4]. The release of proinflammatory cytokines driven by mechanical injury is an important factor in the process of cartilage damage evolved into inflammation and osteoarthritis (OA) [5]. These inflammatory mediators, including TNF-α and IL-1β, are catabolic cytokines involved in the degradation of ECM by promoting the expression of matrix MMPs and ADAMTS [6]. It is reported that IL-1β and TNF-α could markedly reduce the expressions of collagen II (Col2α), aggrecan (AGG) and SOX9 and increase the expression of MMPs [7]. Furthermore, TNF-α increases the cartilage degeneration through the activation of NF-κB and PI3K/AKT [8].

As a HIFs family member, it is reported that HIF-2α is highly expressed in the OA cartilage of mouse and human and plays an important role in inflammation [9,10]. It is demonstrated that NF-κB regulates HIF-2α transcriptional activity basing on a HIF-2α promoter analysis and NF-κB/HIF-2α signaling pathway is tightly associated with the development of OA evolved from articular cartilage damage [11].The NF-κB family is made up of five proteins ubiquitously expressed in mammals: p65 (NF-κB, RelA), c-Rel, RelB, NF-B1 (p105/p50) and NF-B2 (p100/p52). They form homodimers and various heterodimers [12–14]. NF-κB can bind with IκB to form a complex that is maintained in the cytosol. Once IκBs are phosphorylated by IκB kinase (IKK) and degraded by the ubiquitin proteasomes, NF-κB is released from the complex and transfers into the nucleus where it promotes the expressions of the target genes such as HIF-2α [15].

By now, there are no effective drugs and methods to treat the degradation of articular cartilage [16]. In recent years, more and more researchers have turned their attention to the traditional Chinese medicine extracts for cartilage damage and OA treatment. Icariin (ICA) is a typical flavonoid compound originated from *Epimedii Folium* with anti-inflammation, anticancer and bone-protective effects [17]. Our previous studies demonstrate that ICA up-regulates the expressions of HIF-1α and glycolysis key enzymes in chondrocytes, promotes ECM secretion and glycolysis, and maintains the chondrocyte phenotype. The present study, we investigated the effect of ICA on inflammation. Combining with our previous studies, we believe that ICA is one effective candidate drug for the treatment of articular cartilage injury.

## Materials and methods

### Cell vitality assay

Mouse chondrocyte (ADTC5) was a gift from Dr Yang from Jinan University and grown in DMEM medium (Gibco) containing 10% (v/v) FBS (Gibco), 2 mM L-glutamine (Gibco), 100 U/ml penicillin and 100 μg/ml streptomycin (Gibco) at 37°C in an atmosphere with 5% CO_2_. ADTC5 chondrocytes were seeded at 4.0 × 10^3^ cells/well on a 96-well plate and cultured overnight at 37°C. After treated with a range of ICA (0, 10^−7^, 10^−6^ and 10^−5^ mol/l) for 24 h or with TNF-α (20 ng/ml) and 10^−6^ mol/l of ICA for 24 h, cell vitality was determined at 490 nm by the MTS method in accordance with the Cell Titer 96 Aqueous One Solution Viability assay manual (Promega Corporation).

### Western blot analysis

ADTC5 chondrocytes were collected after kinds of treatments and whole-cell lysates were prepared for western blotting in RIPA buffer. Twenty micrograms of proteins were loaded onto an SDS-PAGE system and transferred onto a PVDF membrane (Merck). Membranes were incubated overnight at 4°C with primary antibodies of HIF-2α (#: A01248-1, 1:200, Bosder), p-IKKα/β (#: 86690, 1:1000, Cell Signaling Technology), IKKα/β (#: ab194528, 1:1000, Abcom), NF-κB (#: 8242, 1:1000, Cell Signaling Technology), p-NF-κB (#: 93H1, 1:1000, Cell Signaling Technology), IκBα (#: 14D4, 1:1000, Cell Signaling Technology), p-IκBα (#: 8219, 1:1000, Cell Signaling Technology) and GAPDH (#: 5174, 1:1000, Cell Signaling Technology). Next day, membranes were incubated with secondary anti-rabbit/mouse IgG, HRP-linked antibody (#: 7074/7076, 1:3000, Cell Signaling Technology). After washing with TBST, proteins on the membranes were detected using an electrochemiluminescence (ECL) detection kit (Pierce). The images were captured by ChemiDoc XRS Imaging System (Bio-Rad) and analyzed using Image Lab 5.2.1 software.

### Immunofluorescence

ADTC5 Chondrocytes were seeded on lysine-coated glass coverslips in 24-well plates. After fixed with PFA for 10 min, the chondrocytes were incubated with primary antibody of NF-κB (#: 8242, 1:1000, Cell Signaling Technology) overnight at 4°C. After rinsing, the coverslips were incubated with goat anti-rabbit IgG/Alexa Fluor® 488 secondary antibody (#: ab150077, 1:1000, Abcom) in dark. Finally, the coverslips were mounted with mounting solution containing DAPI (Sigma). Fluorescence images were captured and analyzed by fluorescence microscope (Olympus).

### Quantitative reverse transcription polymerase chain reaction (qRT-PCR)

Total RNA was extracted from ADTC5 chondrocytes using TRIzol reagent (Thermo Fisher Scientific). The reaction procedure was as follows: 85°C 8 s for denaturation and 37°C 15 min for annealing and extension. qRT-PCR was performed with TB Green Premix ExTaq II (#: RR066A, TaKaRa) in qTOWER 2.2 real time PCR sysem (Analytik Jena). β-Actin was used as an internal control. The primer sequences of gene amplification were show in Table 1.

**Table 1:** The primer sequences of gene amplification

Gene	Forward (5′-3′)	Reverse (5′-3′)
SOX9	GTGCAAGCTGGCAAAGTTGA	TGCTCAGTTCACCGATGTCC
Col2α	GGTGAGCCATGATCCGCC	TGGCCCTAATTTTCGGGCATC
AGG	CGTTGCAGACCAGGAGCAAT	CTCGGTCATGAAAGTGGCGG
MMP9	GTACTCGACCTGTACCAGCG	AGAAGCCCCACTTCTTGTCG
ADAMTS5	AAGAGGAGGAGGAGGAGGAGGAG	AATGGTTGTGAGCTGCCGTATGG
β-Actin	GTTGTCGACGACGAGCG	GCACAGAGCCTCGCCTT

### Establishment of mouse articular cartilage defect model

Experimental procedures were carried out according to the protocols approved by Ethics Committee of Guangzhou Red Cross Hospital, Jinan University. The articular cartilage defect model of mice was adapted from previously established procedure [18]. Briefly, C57BL/6 male mice (5 weeks) were anesthetized. The distal femur was exposed and an osteochondral defect with 1 mm in diameter and 2 mm in height was created with a 21 G needle. The defects were implanted with the 3D alginate-gelfoam complexes incorporated with or without ICA. Before the surgery, the 3D alginate-gelfoam complexes were generated as follow: Gelfoams (2 * 2 * 2 mm^3^) were pre-humidified with sterile deionized water, soaked in 4% alginate acid sodium solution containing ICA (10^−6^ mol/l) or no ICA, and then immersed in 102 mmol/l calcium chloride solution. The complexes were then incubated at 37°C for 5 mins and implanted into the cartilage defect. Six mice were used in each group. After feeding with normal food and water for 2 and 6 weeks, the mice were euthanized and the knee joints were isolated from different groups.

### Histology and immunohistochemistry

For histological staining, the samples were fixed, decalcified and embedded. The sections were cut at the thickness of 5 μm. The sections were stained with Hematoxylin & Eosin (H & E) or safranin O/fast green staining (SO). For immunohistochemistry analysis, goat two-step detection kit was used to detect the antigens according to the manufacturer’s instructions (ZSGB-BIO, PV-8000). The sections were incubated with 100 μl diluted primary antibody of rabbit polyclonal anti-NF-κB (#: 8242, 1:200, Cell Signaling Technology) or rabbit polyclonal anti-HIF-2α (#: A01248-1, 1:50, Boster) overnight at 4°C respectively, and then incubated with HRP-conjugated secondary antibody for 1 h at room. The reaction was visualized by incubating the sections with a DAB kit (#: SP-9000, ZSGB-BIO) and hematoxylin for 5 min at room temperature. The photos were taken by an inverted phase contrast microscope (Olympus CKX41-A32PH). The adapted histological parameters originated from the International Cartilage Repair Society (ICRS) II include: (1) matrix staining; (2) subchondral bone; (3) overall assessment. The defect surrounded by the dashed lines was defined as region of interest and employed for analysis. Three blinded readers graded the cartilage sections according to ICRS II parameters and criteria [19].

### Statistical analysis

All data were expressed as mean ± SD and analyzed using SPSS version 17.0 software. The differences between groups were analyzed by Student’s *t*-test or one-way ANOVA. All experiments were performed in triplicate. *P*<0.05 and *P*<0.01 were considered to indicate statistically significant and extreme significant differences.

## Results

### ICA increases ADTC5 chondrocyte vitality and suppresses cartilage degradation

**Figure 1:**
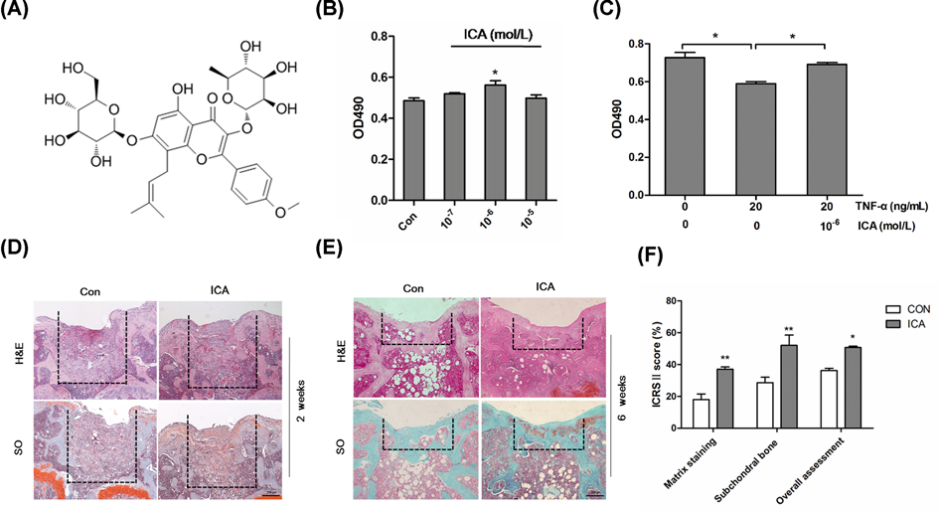
ICA increased chondrocyte vitality and suppressed cartilage degradation (**A**) The chemical formula of ICA. (**B**) Cell vitality of ADTC5 chondrocytes treated for 24 h with the indicated concentrations of ICA was determined by MTS assays. (**C**) Cell vitality of ADTC5 chondrocytes treated for 24 h with TNF-α (20 ng/ml) or/and ICA (10^−6^ mol/l) was determined by MTS assays. (**D** and **E**) ICA suppressed ECM degradation in mouse articular cartilage model. The region surrounded by the dotted lines is the defect. H&E and SO staining showed the defects in mice treated without or with ICA for 2 (D) or 6 (E) weeks; magnification = 100; bar: 200 µm. (**F**) ICRS II score of mice treated for 6 weeks. *P<0.05 and **P<0.01 were regarded as significance and extremely significance, respectively.

ADTC5 chondrocytes were treated with a range of ICA (0, 10^−7^, 10^−6^ and 10^−5^ mol/l) for 24 h. ADTC5 vitality was measured with MTS. The structure of ICA is shown in Figure 1A. The cell vitality increased as the concentration of ICA increased. The highest cell vitality was found at the concentration of 10^−6^ mol/l of ICA (Figure 1B) (*P*<0.05). Basing on our previous study [20] and this, 20 ng/ml of TNF-α was used as an inflammation inducer and 10^−6^ mol/l of ICA was used in the subsequent experiments in chondrocytes. Comparing with control cells, TNF-α treatment significantly inhibited cell vitality. ICA treatment (10^−6^ mol/l) significantly neutralized the decrease of ADTC5 vitality resulted from the TNF-α treatment (Figure 1C).

To evaluate the protective effect of ICA on articular cartilage injury *in vivo*, mouse articular cartilage defect model was established and alginate-gelfoam 3D complexes with (alginate-gelfoam-ICA) or without ICA (alginate-gelfoam-Con) were implanted into the defect area. After implantation for 2 or 6 weeks, the animals were killed and the knee joint samples were collected for histological examination by H & E and SO staining. The areas indicated with dotted line were implanted with alginate–gelfoam 3D complexes (Figure 1D,E). The 3D complexes were completely absorbed after 6 weeks. By H & E and SO staining, we found that the cartilage surface was relatively intact and the subchondral bone was substantial in the alginate-gelfoam-ICA group; while in the alginate-gelfoam-Con group, the cartilage layer was thin, rough, and less matrix staining (Figure 1E). Consistent with these findings, the ICRS II score of matrix staining, subchondral bone and overall assessment in the alginate-gelfoam-ICA group was significantly increased comparing with the alginate-gelfoam-Con group (Figure 1F). These findings indicated that ICA relieved ECM degradation and the destruction of articular cartilage and suppressed cartilage degradation in mice.

### ICA increases the expressions of ECM and ECM regulators in chondrocytes

**Figure 2:**
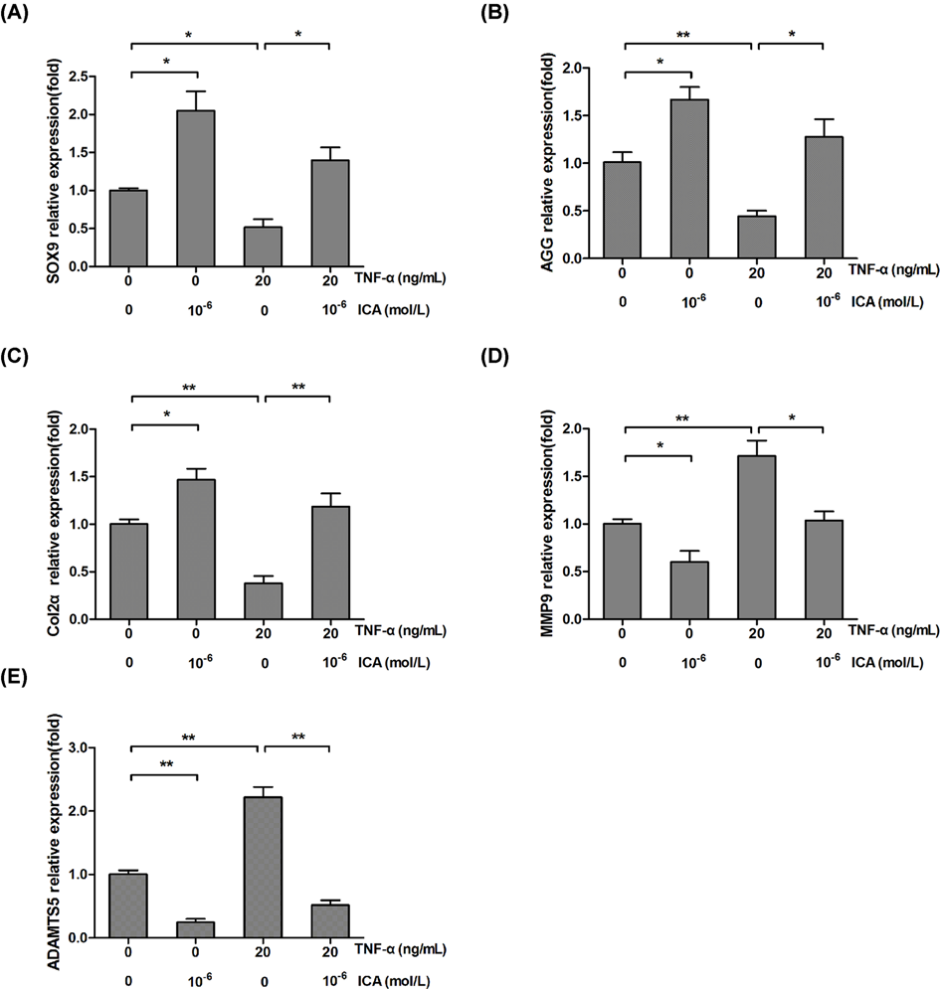
ICA regulated the expression of ECM associated proteins in ADTC5 chondrocytes measured by qRT-PCR (**A–C**) ICA eliminated the down-regulation of TNF-α on the expressions of SOX9 (A), AGG (B) and Col2α (C). (**D** and **E**) ICA eliminated the up-regulation of TNF-α on the expressions of MMP9 (D) and ADAMTS5 (E). Cells were treated with TNF-α (20 ng/ml) or TNF-α and ICA (10^−6^ mol/l) for 24 h. *P<0.05 and **P<0.01 were regarded as significance and extremely significance, respectively.

By SO staining, we could find that ICA obviously increased ECM expression in mouse articular cartilage defect model (Figure 1E). The influence of ICA on the expressions of ECM and ECM regulators in chondrocytes was analyzed by qRT-PCR. SOX9 is an important chondrogenic transcription factor that maintains chondrocyte phenotype and the formation of embryonic cartilage by promoting the expression of Col2α and AGG [21,22]. As shown in Figure 2A–C, the expressions of SOX9, AGG and Col2α were significantly up-regulated by ICA treatment and down-regulated by TNF-α treatment. But, the effect of TNF-α on the expressions of these genes was obviously eliminated by ICA treatment. It has been demonstrated that ECM is degraded by MMPs and ADAMTS families. We found that the expressions of MMP9 and ADAMTS5 were significantly down-regulated by ICA treatment and up-regulated by TNF-α treatment (Figure 2D,E). The mRNAs of MMP9 and ADAMTS5 were increased by 0.8 and 0.6 times, respectively, in TNF-α treatment comparing with control group. However, the effect of TNF-α on the expressions of MMP9 and ADAMTS5 was obviously abolished by ICA treatment. These results suggest that ICA could increase ECM in chondrocytes by promoting ECM synthesis and inhibiting ECM degradation.

### ICA attenuates TNF-α-driven inflammation and NF-κB/HIF-2α signal pathway *in vitro*

**Figure 3:**
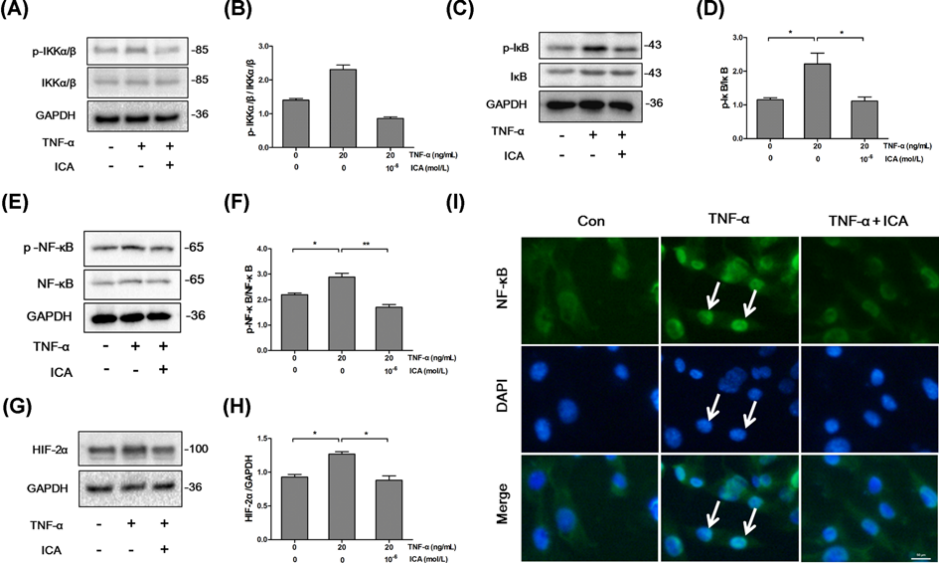
ICA inhibited NF-κB/HIF-2α signal pathway in ADTC5 chondrocytes (**A** and **B**) ICA attenuated the increase of p-IKKα/β/IKKα/β ratio induced by TNF-α treatment. Chondrocytes were treated with TNF-α or TNF-α and ICA for 24 h. The expressions of p-IKKα/β and /IKKα/β were checked by Western blot (A and B) indicated p-IKKα/β/IKKα/β ratio. (**C** and **D**) ICA attenuated the increase of p-IκB/IκB ratio induced by TNF-α treatment. Chondrocytes were treated with TNF-α or TNF-α and ICA for 24 h. The expression of p-IκB and IκB was checked by Western blot (C and D) indicated p-IκB/IκB ratio. (**E** and **F**) ICA attenuated the increase of p-NF-κB/NF-κB ratio induced by TNF-α treatment. Chondrocytes were treated with TNF-α or TNF-α and ICA for 24 h. The expressions of p-NF-κB and NF-κB were checked by Western blot (E and F) indicated p-NF-κB/NF-κB ratio. (**G** and **H**) ICA attenuated the increase of HIF-2α expression induced by TNF-α treatment. Chondrocytes were treated with TNF-α or TNF-α and ICA for 24 h. HIF-2α expressions was checked by Western blot (G and H) indicated the relative expression of HIF-2α against GAPDH. (**I**) ICA attenuated NF-κB nucleic localization induced by TNF-α treatment; magnification = 200; bar: 50 µm; *P<0.05 and **P<0.01 were regarded as significance and extremely significance, respectively.

It is reported that HIF-2α is highly expressed in OA cartilage and is regulated by NF-κB. NF-κB/HIF-2α signaling pathway is tightly associated with the development of OA evolved from articular cartilage damage and NF-κB is key mediator in the signaling pathway that activates the cascade of inflammation in chondrocytes [15]. The crucial steps in the activation of NF-κB are the phosphorylation of IκB by IκB kinase complex and the nuclear translocation of NF-κB. To clarify the effect of ICA on NF-κB/HIF-2α signaling pathway, the ratios of p-IKKα/β/IKKα/β, p-IκB/IκB, p-NF-κB/NF-κB and HIF-2α expression were measured in ADTC5 chondrocytes. As shown in Figure 3A–H, the ratios of p-IKKα/β/IKKα/β, p-IκB/IκB and p-NF-κB/NF-κB were significantly up-regulated by TNF-α treatment, respectively. But ICA treatment neutralized the increases of p-IKKα/β/IKKα/β, p-IκB/IκB and p-NF-κB/NF-κB induced by TNF-α treatment, respectively. The expression of HIF-2α was dramatically up-regulated by TNF-α treatment comparing with control (*P*<0.05), but it was significantly decreased by ICA treatment (*P*<0.05) (Figure 3G,H). Since the nucleic translocation of NF-κB is needed for the activation of NF-κB/HIF-2α signaling pathway, NF-κB localization in ADTC5 chondrocytes was investigated by immunofluorescence staining. Accompanied with the significance increase of NF-κB (green) in TNF-α-treated ADTC5 chondrocytes, more NF-κB transferred to nuclei comparing with control cells (Figure 3I). However, ICA treatment inhibited the increase and nucleic translocation of NF-κB induced by TNF-α treatment (Figure 3I). These results suggest that NF-κB/HIF-2α signaling pathway was activated by TNF-α treatment and ICA could neutralize the inflammation effect induced by TNF-α in ADTC5 chondrocytes.

### ICA suppressed the NF-κB/HIF-2α signal pathway *in vivo*

**Figure 4:**
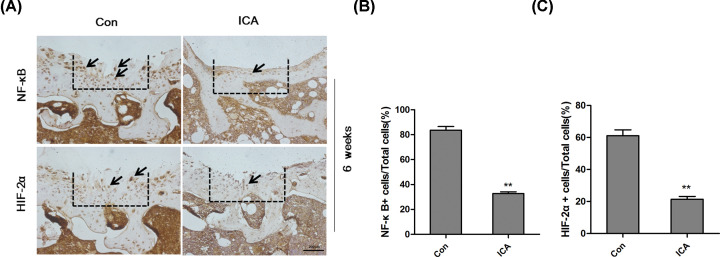
ICA suppressed the NF-κB/HIF-2α signal pathway in mice (**A**) Immunohistochemical staining for HIF-2α and NF-κB in mouse defects; magnification = 100; bar: 200 µm. (**B** and **C**) The percentage of NF-κB positive cells (B) and HIF-2α positive cells. The regions surrounded by the dotted lines were the defects. **P<0.01 were regarded as significance and extremely significance.

In chondrocytes, we found that the NF-κB/HIF-2α signal pathway was activated by TNF-α and inhibited by ICA. Furthermore, we checked the influence of ICA on NF-κB/HIF-2α signal pathway *in vivo* using mouse articular cartilage defect model. The dotted line indicated the area where the 3D alginate-gelfoam complexes were implanted. Since 3D complexe was completely absorbed after 6 weeks, hereby immunohistochemistry and quantitation analysis were only applied on 6 week-tissues. The positive cells of HIF-2α and NF-κB were significant less in the defect region implanted with the complexes containing ICA (alginate-gelfoam-ICA) than that of control (alginate-gelfoam-Con) (Figure 4A–C). These data indicated that ICA could relieve inflammation *in vivo* as well by inhibiting the NF-κB/HIF-2α signal pathway.

## Discussion

Traditional Chinese medicine is becoming more and more highly regarded in recent years because of the advantages of rich sources, low prices, and stable efficacy. Researchers have been focused on the protective effects of the active ingredients of Chinese medicine on cartilage damage [23]. According to the literature, ICA is expected to replace growth factors for the treatment of various cartilage injuries [24]. Our previous studies demonstrate that ICA protects cartilage chondrocyte vitality by promoting glycolysis and HIF-1α [25,26]. In the present study, we demonstrated that ICA protects cartilage chondrocyte vitality by neutralizing the inflammation induced by NF-κB/HIF-2α signal pathway. Basing on this and our previous studies, we demonstrate that ICA is an effectively potential ingredient against cartilage injury.

It is reported that TNF-α plays an vital role in the development of OA from chondrocyte inflammation that usually acts as a trigger initiating an imbalance between degradation and synthesis of articular cartilage [27,28]. The inflammatory response stimulated by TNF-α in chondrocytes is closely involved in the NF-κB/HIF-2α signaling pathway [29,30]. Since HIF-2α is activated as a target of NF-κB upon the treatment of TNF-α [31], TNF-α (20 ng/ml) was used to establish a chondrocyte inflammation model in the present study. Western blot results showed that the ratios of p-IKKα/β/IKKα/β, p-IκB/IκB and p-NF-kB/NF-κB and HIF-2α expression in TNF-α-treatment chondrocytes were significantly higher than that in chondrocytes without TNF-α-treatment (control). The immunofluorescent results indicated that more NF-κB transferred to nuclei after the chondrocytes were treated with TNF-α. As the downstream targets of NF-κB/HIF-2α signal pathway [32,33] and the main performers of cartilage degradation and destruction [34], the expressions of MMP9 and ADMTS5 were up-regulated in the chondrocytes that were treated with TNF-α. In contrast, the expressions of chondrocyte specific markers including SOX9, AGG and Col2α were down-regulated. These results suggest that TNF-α, an important inflammatory factor, can promote cartilage damage by activating NF-κB/HIF-2α signal pathway. It is amazing that ICA neutralizes the inflammation induced by TNF-α in chondrocytes. ICA treatment attenuated the expression of HIF-2α, MMP9 and ADMTS5, NF-κB nucleic translocation and the ratios of p-IKKα/β/IKKα/β, p-IκB/IκB and p-NF-kB/NF-κB. ICA treatment also promoted the expressions of SOX9, AGG and Col2α.

Mouse articular cartilage defect is a popular inflammatory and cartilage degradation model. The cartilage inflammation and degradation induced by articular cartilage defect model in mice are corresponded to that induced by TNF-α in chondrocytes. The influence of ICA on ECM synthesis and the expression of NF-κB and HIF-2α *in vivo* was investigated with mouse articular cartilage defect model in the present study. The alginate–gelfoams complex, a slow-release and absorbable gelatin, without or with ICA was implanted into the defects. ICRS II score and ECM synthesis were significantly higher in the mice implanted with the alginate–gelfoam complexes containing ICA than in the mice implanted with the alginate–gelfoam complexes non-containing ICA. However, the expressions of NF-κB and HIF-2α were significantly higher in the mice implanted with the alginate–gelfoam complexes non-containing ICA than in the mice implanted with the alginate–gelfoam complexes containing ICA. These results demonstrated that ICA significantly inhibited the cartilage degradation, inflammation and NF-κB/HIF-2α signaling pathways *in vivo*.

Our previous studies demonstrated that ICA increases the vitality of chondrocytes and ECM synthesis by promoting HIF-1α expression and anaerobic glycolysis [25,26]. It is reported that HIF-2α and HIF-1α has the opposite effect in chondrocytes and high HIF-2α expression may lead to the destruction and degradation of cartilage. In the present study, we demonstrated that ICA protects chondrocytes from inflammation by inhibiting the activation of NF-κB/HIF-2α signaling pathways through the neutralization of IKKs and IκB phosphorylation. Except inhibiting IKKs and IκB phosphorylation, ICA inhibits STAT3 signal pathway [35]. It is reported that NF-κB signaling pathway can be regulated by STAT3 pathway [36]. The influence of ICA on the potential cross-talk between NF-κB and STAT3 needs to be investigated in future.

**Figure 5:**
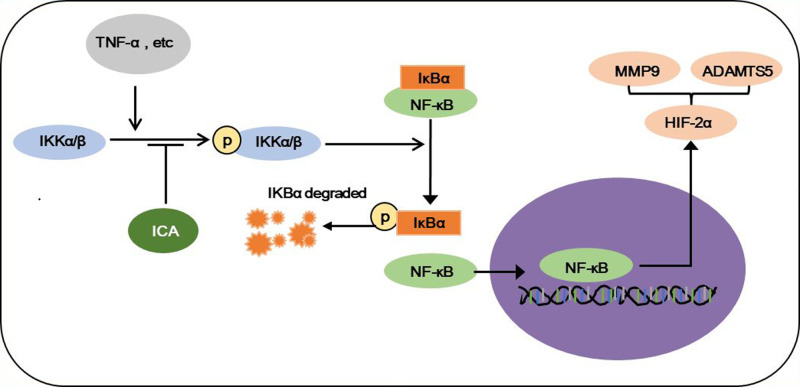
Schematic diagram of the protection of ICA on chondrocytes ICA protects chondrocytes from inflammation induced by TNF-α through inhibiting the phosphorylation of IKKα/β, the activation of NF-κB/HIF-2α and the nuclear translocation of NF-κB. Subsequently, ICA inhibits ECM degradation by decreasing the production of MMPs and ADMTS5.

In conclusion, ICA protects chondrocytes from inflammation by inhibiting the activation of NF-κB/HIF-2α signal pathway. The main results can be concluded in Figure 5. ICA inhibited the phosphorylation of IKKs, IκB and NF-κB and the expression of HIF-2α induced by TNF-α treatment. As a result, ICA inhibited the expressions of MMP9 and ADAMTS5, suppressed cartilage degradation and increased chondrocyte vitality. Combing this and our previous studies, we demonstrate that ICA could be a potential molecule for the clinical treatment of OA by suppressing cartilage degradation via its effect on anaerobic glycolysis and inflammation.

## Data Availability

All data associated with the present study are included in this published article or are available from the corresponding author on reasonable request.

## Abbreviations

AGG: aggrecan
Col2α: collagen II
ECM: extracellular matrix
ICA: icariin
IKK: IκB kinase
MMP: metalloproteinase

## References

[1] Makris E.A., Gomoll A.H., Malizos K.N., Hu J.C. and Athanasiou K.A. (2015) Repair and tissue engineering techniques for articular cartilage. Nat. Rev. Rheumatol. 11, 21–34 10.1038/nrrheum.2014.157 25247412 PMC4629810

[2] Tang X., Wang S., Zhan S., Niu J., Tao K., Zhang Y. et al. (2016) The Prevalence of Symptomatic Knee Osteoarthritis in China: Results From the China Health and Retirement Longitudinal Study. Arthritis Rheumatol. (Hoboken, N.J.) 68, 648–653 10.1002/art.39465 26474054

[3] Krishnan Y. and Grodzinsky A.J. (2018) Cartilage diseases. Matrix Biol.: J. Int. Soc. Matrix Biol. 71, 51–69 10.1016/j.matbio.2018.05.005 PMC6146013 29803938

[4] Chen C., Zhu Z., Hu N., Liang X. and Huang W. (2020) Leonurine Hydrochloride Suppresses Inflammatory Responses and Ameliorates Cartilage Degradation in Osteoarthritis via NF-κB Signaling Pathway. Inflammation 43, 146–154 10.1007/s10753-019-01104-z 31659586

[5] Wojdasiewicz P., Poniatowski Ł.A. and Szukiewicz D. (2014) The role of inflammatory and anti-inflammatory cytokines in the pathogenesis of osteoarthritis. Mediators Inflamm. 2014, 561459 10.1155/2014/561459 24876674 PMC4021678

[6] Chen C., Xie J., Rajappa R., Deng L., Fredberg J. and Yang L. (2015) Interleukin-1β and tumor necrosis factor-α increase stiffness and impair contractile function of articular chondrocytes. Acta Biochim. Biophys. Sin. (Shanghai) 47, 121–129 10.1093/abbs/gmu116 25520178 PMC4351355

[7] Ouyang Y., Wang W., Tu B., Zhu Y., Fan C. and Li Y. (2019) Overexpression of SOX9 alleviates the progression of human osteoarthritis in vitro and in vivo. Drug Des. Dev. Ther. 13, 2833–2842 10.2147/DDDT.S203974 PMC6698167 31496660

[8] Li W., Wang W., Liu L., Qu R., Chen X., Qiu C. et al. (2019) GDF11 antagonizes TNF-α-induced inflammation and protects against the development of inflammatory arthritis in mice. FASEB J. 33, 3317–3329 10.1096/fj.201801375RR 30407878

[9] Bracken C.P., Whitelaw M.L. and Peet D.J. (2003) The hypoxia-inducible factors: key transcriptional regulators of hypoxic responses. Cell. Mol. Life Sci.: CMLS 60, 1376–1393 10.1007/s00018-003-2370-y 12943226 PMC11138858

[10] Semenza G.L. (2000) HIF-1 and human disease: one highly involved factor. Genes Dev. 14, 1983–1991 10950862

[11] Murahashi Y., Yano F., Kobayashi H., Makii Y., Iba K., Yamashita T. et al. (2018) Intra-articular administration of IκBα kinase inhibitor suppresses mouse knee osteoarthritis via downregulation of the NF-κB/HIF-2α axis. Sci. Rep. 8, 16475 10.1038/s41598-018-34830-9 30405206 PMC6220282

[12] Jimi E. and Ghosh S. (2005) Role of nuclear factor-kappaB in the immune system and bone. Immunol. Rev. 208, 80–87 10.1111/j.0105-2896.2005.00329.x 16313342

[13] De Luca F. (2016) Role of Nuclear Factor Kappa B (NF-κB) in Growth Plate Chondrogenesis. Pediatric Endocrinol. Rev.: PER 13, 720–730 27464415

[14] Kobayashi H., Chang S.H., Mori D., Itoh S., Hirata M., Hosaka Y. et al. (2016) Biphasic regulation of chondrocytes by Rela through induction of anti-apoptotic and catabolic target genes. Nat. Commun. 7, 13336 10.1038/ncomms13336 27830706 PMC5109547

[15] Hayden M.S. and Ghosh S. (2014) Regulation of NF-κB by TNF family cytokines. Semin. Immunol. 26, 253–266 10.1016/j.smim.2014.05.004 24958609 PMC4156877

[16] Glasson S.S., Askew R., Sheppard B., Carito B., Blanchet T., Ma H.L. et al. (2005) Deletion of active ADAMTS5 prevents cartilage degradation in a murine model of osteoarthritis. Nature 434, 644–648 10.1038/nature03369 15800624

[17] Huang H., Zhang Z.F., Qin F.W., Tang W., Liu D.H., Wu P.Y. et al. (2019) Icariin inhibits chondrocyte apoptosis and angiogenesis by regulating the TDP-43 signaling pathway. Mol. Genet. Genomic Med. 7, e00586 10.1002/mgg3.586 30734541 PMC6465670

[18] Eltawil N.M., De Bari C., Achan P., Pitzalis C. and Dell'accio F. (2009) A novel in vivo murine model of cartilage regeneration. Age and strain-dependent outcome after joint surface injury. Osteoarthritis Cartilage 17, 695–704 10.1016/j.joca.2008.11.003 19070514 PMC2706394

[19] Mainil-Varlet P., Van Damme B., Nesic D., Knutsen G., Kandel R. and Roberts S. (2010) A new histology scoring system for the assessment of the quality of human cartilage repair: ICRS II. Am. J. Sports Med. 38, 880–890 10.1177/0363546509359068 20203290

[20] Li S., Yang X., Feng Z., Wang P., Zhu W. and Cui S. (2018) Catalase Enhances Viability of Human Chondrocytes in Culture by Reducing Reactive Oxygen Species and Counteracting Tumor Necrosis Factor-α-Induced Apoptosis. Cell. Physiol. Biochem.: Int. J. Exp. Cell. Physiol. Biochem. Pharmacol. 49, 2427–2442 10.1159/000493841 30261500

[21] Henry S.P., Liang S., Akdemir K.C. and de Crombrugghe B. (2012) The postnatal role of Sox9 in cartilage. J. Bone Mineral Res.: Off. J. Am. Soc. Bone Mineral Res. 27, 2511–2525 10.1002/jbmr.1696 PMC3502666 22777888

[22] Hattori T., Müller C., Gebhard S., Bauer E., Pausch F., Schlund B. et al. (2010) SOX9 is a major negative regulator of cartilage vascularization, bone marrow formation and endochondral ossification. Development 137, 901–911 10.1242/dev.045203 20179096

[23] Li L., Liu H., Shi W., Liu H., Yang J., Xu D. et al. (2017) Insights into the Action Mechanisms of Traditional Chinese Medicine in Osteoarthritis. Evidence-Based Complement. Alternative Med.: eCAM 2017, 5190986 10.1155/2017/5190986 PMC5292158 28203259

[24] Li D., Yuan T., Zhang X., Xiao Y., Wang R., Fan Y. et al. (2012) Icariin: a potential promoting compound for cartilage tissue engineering. Osteoarthritis Cartilage 20, 1647–1656 10.1016/j.joca.2012.08.009 22917745

[25] Wang P., Xiong X., Zhang J., Qin S., Wang W., Liu Z. (2020) Icariin increases chondrocyte vitality by promoting hypoxia-inducible factor-1α expression and anaerobic glycolysis). The Knee 27, 18–25 10.1016/j.knee.2019.09.012 31883860

[26] Wang P., Zhang F., He Q., Wang J., Shiu H.T., Shu Y. et al. (2016) Flavonoid Compound Icariin Activates Hypoxia Inducible Factor-1α in Chondrocytes and Promotes Articular Cartilage Repair. PLoS One 11, e0148372 10.1371/journal.pone.0148372 26841115 PMC4739592

[27] Kapoor M., Martel-Pelletier J., Lajeunesse D., Pelletier J.P. and Fahmi H. (2011) Role of proinflammatory cytokines in the pathophysiology of osteoarthritis. Nat. Rev. Rheumatol. 7, 33–42 10.1038/nrrheum.2010.196 21119608

[28] Bay-Jensen A.C., Henrotin Y., Karsdal M. and Mobasheri A. (2016) The Need for Predictive, Prognostic, Objective and Complementary Blood-Based Biomarkers in Osteoarthritis (OA). EBioMedicine 7, 4–6 10.1016/j.ebiom.2016.05.004 27322444 PMC4909481

[29] Liacini A., Sylvester J., Li W.Q., Huang W., Dehnade F., Ahmad M. et al. (2003) Induction of matrix metalloproteinase-13 gene expression by TNF-alpha is mediated by MAP kinases, AP-1, and NF-kappaB transcription factors in articular chondrocytes. Exp. Cell Res. 288, 208–217 10.1016/S0014-4827(03)00180-0 12878172

[30] Tak P.P. and Firestein G.S. (2001) NF-kappaB: a key role in inflammatory diseases. J. Clin. Invest. 107, 7–11 10.1172/JCI11830 11134171 PMC198552

[31] Chen D., Lu D., Liu H., Xue E., Zhang Y., Shang P. et al. (2019) Pharmacological blockade of PCAF ameliorates osteoarthritis development via dual inhibition of TNF-α-driven inflammation and ER stress. EBioMedicine 50, 395–407 10.1016/j.ebiom.2019.10.054 31735552 PMC6921217

[32] Yang S., Kim J., Ryu J.H., Oh H., Chun C.H., Kim B.J. et al. (2010) Hypoxia-inducible factor-2alpha is a catabolic regulator of osteoarthritic cartilage destruction. Nat. Med. 16, 687–693 10.1038/nm.2153 20495569

[33] Saito T., Fukai A., Mabuchi A., Ikeda T., Yano F., Ohba S. et al. (2010) Transcriptional regulation of endochondral ossification by HIF-2alpha during skeletal growth and osteoarthritis development. Nat. Med. 16, 678–686 10.1038/nm.2146 20495570

[34] Chern C.M., Zhou H., Wang Y.H., Chang C.L., Chiou W.F., Chang W.T. et al. (2020) Osthole ameliorates cartilage degradation by downregulation of NF-κB and HIF-2α pathways in an osteoarthritis murine model. Eur. J. Pharmacol. 867, 172799 10.1016/j.ejphar.2019.172799 31765607

[35] Chi L., Gao W., Shu X. and Lu X. (2014) A natural flavonoid glucoside, icariin, regulates Th17 and alleviates rheumatoid arthritis in a murine model. Mediators Inflamm. 2014, 392062 10.1155/2014/392062 25374443 PMC4211316

[36] Hoesel B. and Schmid J.A. (2013) The complexity of NF-κB signaling in inflammation and cancer. Mol. Cancer 12, 86 10.1186/1476-4598-12-86 23915189 PMC3750319

